# The Distribution Pattern and Species Richness of Scorpionflies (Mecoptera: Panorpidae)

**DOI:** 10.3390/insects14040332

**Published:** 2023-03-29

**Authors:** Jian Su, Wanjing Liu, Fangcheng Hu, Panpan Miao, Lianxi Xing, Yuan Hua

**Affiliations:** 1 College of Life Sciences, Northwest University, Xi’an 710069, China; 2 Shaanxi Key Laboratory for Animal Conservation, Northwest University, Xi’an 710069, China; 3 Key Laboratory of Resource Biology and Biotechnology in Western China (Northwest University), Ministry of Education, Xi’an 710069, China

**Keywords:** Panorpidae, Mecoptera, distribution pattern, species richness, MaxEnt

## Abstract

**Simple Summary:**

Biodiversity and distribution patterns are important factors for ecological and biogeographical studies. However, half of the high species diversity areas are found in mountainous regions, which have been heavily impacted by climate change, land use changes, and habitat fragmentation. This makes mountain habitats and biodiversity more vulnerable than before. Scorpionflies, as ecological indicators with a narrow distribution, low-temperature preference, and weak migration ability, are ideal animals for studying the impact of climate change on insect distribution. Predicting the distribution of suitable habitats for species in different periods can help clarify the impact of climate change on species distribution and provide guidance for the conservation of endangered species.

**Abstract:**

The uneven distribution of species diversity on earth, with mountainous regions housing half of the high species diversity areas, makes mountain ecosystems vital to biodiversity conservation. The Panorpidae are ecological indicators, ideal for studying the impact of climate change on potential insect distribution. This study examines the impact of environmental factors on the distribution of the Panorpidae and analyzes how their distribution has changed over three historical periods, the Last Interglacial (LIG), the Last Glacial Maximum (LGM), and Current. The MaxEnt model is used to predict the potential distribution area of Panorpidae based on global distribution data. The results show that precipitation and elevation are the primary factors affecting species richness, and the suitable areas for Panorpidae are distributed in southeastern North America, Europe, and southeastern Asia. Throughout the three historical periods, there was an initial increase followed by a decrease in the area of suitable habitats. During the LGM period, there was a maximum range of suitable habitats for cool-adapted insects, such as scorpionflies. Under the scenarios of global warming, the suitable habitats for Panorpidae would shrink, posing a challenge to the conservation of biodiversity. The study provides insights into the potential geographic range of Panorpidae and helps understand the impact of climate change on their distribution.

## 1. Introduction

The biodiversity and distribution patterns are fundamental to ecological and biogeographic studies of living organisms [1]. However, the distribution of species diversity on earth is uneven, with half the high species diversity areas located in mountainous regions [2]. Mountain ecosystems are distinctive and greatly impact biodiversity preservation [1]. However, the mountain ecosystems have suffered significant deterioration owing to climate change, land use change, and habitat fragmentation, making mountain habitats and biodiversity more vulnerable than before.

Panorpidae, the largest family of Mecoptera, are commonly called scorpionflies because of the shape of the male genitalia. Scorpionflies are weak fliers, preferring cool, humid habitats, and are vulnerable to high temperatures. They generally inhabit mountainous regions higher than 1000 m and are unable to migrate over a long distance, forming multiple “sky islands” in high-altitude mountain regions [3]. This kind of mountain species with a narrow distribution, low-temperature preference, and weak migration ability is ideal material for studying the impact of climate change on the potential distribution of insects [4], such as neotenic net-winged beetles, sandflies, and carabid beetles. Liu et al. used the MaxEnt and the random forest models to investigate the potential geographic distribution and environmental adaptability of red fireflies in China [5]. Townsend et al. used the niche simulation method to analyze the geographic and ecological distribution of three types of sand flies in South America, as well as the impact of climate change [6]; Liu et al. used the MaxEnt model to predict the impact of current climate change on the distribution of steppe beetles, which facilitated follow-up diversity studies [7].

Scorpionflies, important components of mountain ecological systems, are significant ecological indicators. Thus, determining the potential geographic range of the Panorpidae is crucial for speculating the range of undiscovered new species, studying its relationship with the environment, and conserving the biodiversity. It can also provide a basis for taxonomic and phylogenetic studies from the perspective of geography. In recent years, research on Panorpidae mainly focused on taxonomy, biogeography, behavior, and evolutionary biology [4,8,9,10], while the ecological study is lagging behind. Dvorak (2018) examined the species richness of Panorpidae at various elevations within the Lagodekhi Protected Areas and found that the diversity of Panorpidae exhibited a monotonically declining trend with increasing elevation [11]. According to Wang et al., the number of Panorpidae species peaked in the intermediate elevation zone of the Qinling Mountains and displayed a unimodal pattern with increasing height [12]. Habitat size and location vary with climate change, prompting migration and dispersal of populations [13]. Predicting the distribution of suitable habitats for species in different periods can help clarify the impact of climate change on species distribution. At the same time, it is an important guide for conserving endangered species and delineating natural preserve areas [14].

After the Last Glacial Maximum in the Pleistocene, the climate change led to the continuous migration, diffusion, and isolation of populations, which shaped the distribution pattern of today’s species [15]. Global temperature dropped during the ice age, pushing certain cold-adapted montane species to migrate to lower latitudes and altitudes, thereby expanding their habitats [16,17,18]. After glacial periods, they retreated back to the mountains and high latitudes, causing their distribution patterns to shrink significantly.

With global warming, the habitats and ecological niches of montane species are becoming narrower. Predicting the suitable areas for mountain species in different periods can help understand the changing trend of the migration and dispersal of the cold-adapted mountain species. The geographical distribution of species reflects their ecological adaptability, diffusibility, and evolutionary history. Understanding the spatial distribution pattern of species is one of the core issues of biogeography and ecology [7,19,20,21]. Environmental and climatic factors are primary determinants leading to biological metabolism and reproduction [22]. Therefore, the geographical distribution of species is closely related to climatic factors [23,24,25]. Ecological niche models (ENMs) provide a means of characterizing the spatial distribution of suitable conditions for species, and have been applied to establish models of specific environmental conditions that meet the ecological requirements of species [26,27]. ENMs predict habitats with higher adaptability to draw a probability map suitable for species distribution [28]. The maximum entropy (MaxEnt) model is widely used in many fields of ecological and biogeography research, such as protecting endangered species, monitoring and managing the natural environment [29], maintaining biodiversity, and predicting species distribution [30]. These studies play a guiding role in understanding the potential distribution of species and formulating conservation measures for endangered species.

In this study, the MaxEnt method was used to predict the potential distribution area of Panorpidae according to the global distribution data. The purposes of this study are as follows: (1) to investigate the impact of climatic factors on the distribution of Panorpidae and the relationship between Panorpidae species richness and environmental factors; (2) to examine how the distribution of Panorpidae changed over three historical periods, the Last Interglacial (LIG), the Last Glacial Maximum (LGM), and 1970–2000 (Current); and (3) to analyze the impacts of global warming on the suitable habitat of Panorpidae.

## 2. Materials and Methods

### 2.1. Data Resources

The occurrence records of Panorpidae were gathered from the following sources: the Global Biodiversity Information Facility (GBIF, https://www.gbif.org/ (accessed on 5 June 2022)), field collection records, and published papers and theses [31,32,33,34,35,36]. A database of 17,847 distribution records for 537 Panorpidae species was obtained. Spatial autocorrelation analysis was performed using the R package “spThin” to lessen the effects of overfitting on the model caused by the clustering of species distributions. This study picked up 1100 valid records for analysis after deleting duplicate and invalid records.

Nineteen bioclimatic variables were obtained from the worldclim database (http://www.worldclim.org (accessed on 5 June 2022)) for the Last Interglacial (LIG), the Last Glacial Maximum (LGM), and 1970–2000 (Current) periods with a resolution of 2.5 (4.3 km × 4.3 km) [37]. Elevation data were also obtained from the worldclim database (http://www.worldclim.org (accessed on 5 June 2022)) with a resolution of 2.5 (4.3 km × 4.3 km). Climate data with a resolution of 2.5 (4.3 km × 4.3 km) and the CCSM4 climate model are utilized for climate prediction. Representative concentration paths (RCPs) are defined by the Intergovernmental Panel on Climate Change (IPCC) as the trajectories of four greenhouse gas concentrations [38]. RCPs come in four different varieties: RCP2.6, RCP4.5, RCP6.0, and RCP8.5. They represent the CO_2_ concentrations of 490, 650, 850, and 1370 mL/m^3^ and the net radiation intensities of 2.6, 4.5, 6.0, and 8.5 W/m^2^ at the end of 2100, respectively [39,40]. In this study, the suitable distribution of Panorpidae was simulated under RCP4.5 from 2061 to 2080. Finally, the globe base map was obtained from the National Basic Geographic Information System (http://www.diva-gis.org/ (accessed on 5 June 2022)).

### 2.2. Identifying Bioclimatic Variables

To reduce the impact of strongly correlated environmental variable data on the prediction outcomes, 19 bioclimatic variables were subjected to principal component analysis (PCA) using the “cor” function in R 4.2.0. One variable was chosen for actual analysis from each set of highly cross-correlated variables in the correlation analysis results (R > 0.8) [41]. Different environmental variables were taken into account, and the most important factors for evaluation and prediction were chosen [42,43]. Contribution rates of 19 bioclimatic variables were calculated using the MaxEnt model (Table 1). The test set consisted of 10% of the total distribution points, while the training set consisted of 90% of the distribution points. The model was programmed to run ten times. Six bioclimatic variables were chosen by the Jackknife test (contribution rate > 0.4) to assess how the distribution of Panorpidae is influenced by climate change.

### 2.3. Analysis of Species Richness

The patterns of species richness were visualized using a grid of size 1 × 1 (~100 km × 100 km) in ArcGIS 10.5 (ESRI Inc., Redlands, CA, USA). Each species’ presence (1) or absence (0) matrix was built using DIVA-GIS 7.5 [44]. The aforementioned matrix was input into EstimateS 9.1 software to obtain a cumulative curve of species constructed based on the bootstrap method, which was used to evaluate the accuracy of the collection of species numbers in the worldwide region [45]. In ArcGIS 10.5, we computed the average of 19 bioclimatic variables and elevation data 4.5 km × 4.5 km grid cells to acquire the variable values in a 1° grid. To ensure the normality of the data and species richness, Bio5, Bio7, Bio10, Bio14, Bio17, and Bio19 were square root transformed, while Bio2, Bio3, Bio12, Bio13, Bio15, and Bio16 were log transformed. The remaining variables were not modified. The entire set of data was then normalized using z-scores.

The association between species richness and each environmental factor was examined using the ordinary least squares (OLS) method. The probability of making a type I error during regression analysis may arise while spatial autocorrelation was ignored. Therefore, the spatial error simultaneous autoregressive analysis model (SARerr) was established using the R package “spdep” to address the spatial autocorrelation in the residuals of the regression model.

### 2.4. Model Evaluation Index

In order to make experiment results reliable, the performance of the MaxEnt model must be evaluated. TSS (the true skill statistic) and AUC (Area Under the Curve) were used as model performance evaluation metrics because their combination can more accurately assess the model’s performance [46,47]. The AUC value was obtained from MaxEnt [40], while the ROC curve is illustrated with the true-positive rate (sensitivity) as the ordinate and the false-positive rate (specificity) of various thresholds as the abscissa. The region encircled by the curve and abscissa was used to determine the AUC value [48]. The TSS value was specificity + sensitivity − 1 [49]. The model’s performance can be classified as failing (0.5–0.6), bad (0.6–0.7), fair (0.7–0.8), good (0.8–0.9), or excellent (0.9–1) based on the AUC value [47]. The model performance can alternatively be generally categorized as failing (<0.4), bad (0.4–0.55), fair (0.55–0.7), good (0.7–0.85), or exceptional (0.85–1) based on the TSS value [46].

### 2.5. Model Setting

The real existence point operation model was used in this analysis, with 10,000 verification points randomly established. The MaxEnt model was modified as follows to screen the ideal model: (1) the multipliers for regularization were successively set to 1, 2, 5, 10, 15, and 20 [27,50,51]; (2) ten cross-validations were selected as the repeat-run type [52]; (3) cloglog was chosen as the output file [53]; (4) the resolutions of the 19 biological environment variables were set to 2.5, 5.0, and 10.0 arc minutes. The regularization multiplier was set to 1, the random test percentage was set to 25, the application threshold rule was set to equal training sensitivity and specificity, the replication run type was cross-validation, and the output format was clog log. Other options were set by default. The MaxEnt model was repeated ten times in this manner. Additionally, a 2.5 min resolution for the 19 environmental parameters was chosen.

### 2.6. Predicting the Appropriate Panorpidae Habitat under Climate Change

The “ASCII to Raster” tool in ArcGIS10.5 was used to convert the *.asc file produced by MaxEnt model software into a raster file. The outcomes were then divided into four categories using the “reclassification” tool and the “natural discontinuity grading” approach; the discontinuity values were 0.2, 0.4, 0.6, and 1, respectively [54]. The Panorpidae worldwide suitability distribution map was then created. Consequently, the Panorpidae’s suitable survival area was divided into four grades: 20%, denoting that it is not suitable for Panorpidae’s survival; 20–40%, denoting the low suitable survival area of Panorpidae; 40–60%, denoting the middle suitable field of Panorpidae; and >60%, denoting the highly suitable survival area of Panorpidae [10].

## 3. Results

### 3.1. Relationships between Species Richness and Environmental Variables

**Figure 1:**
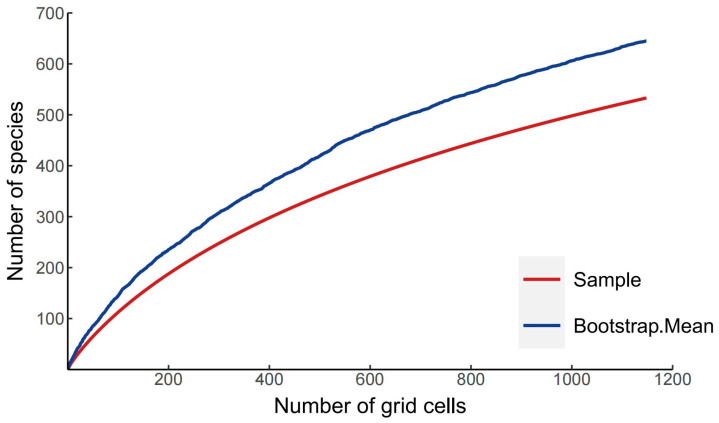
Species accumulation curves for Panorpidae based on bootstrap mean values.

**Figure 2:**
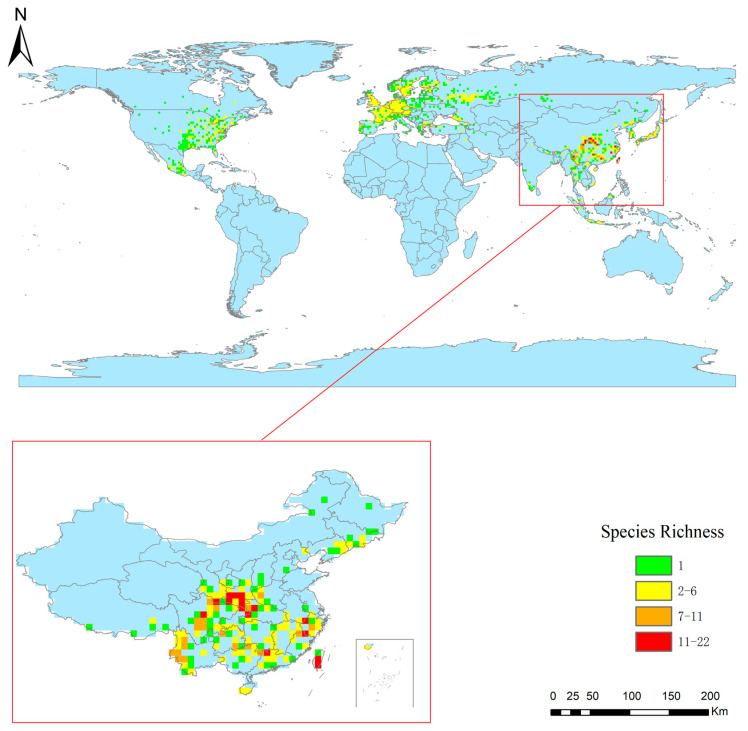
Pattern of species richness on the 1° grid for the Panorpidae.

The results of the species accumulation curve revealed that there were 537 species of Panorpidae that had been collected in the database, compared to 646 species that had been obtained using the bootstrap mean method, a ratio of 83.12%, indicating that the majority of the species had been collected (Figure 1). Furthermore, the patterns of spatial species richness were uneven, with a small number of species found in Europe, a moderate number of species in North America, and many more found in central and southeast China. Specifically, the Qinling Mountains, the Bashan Mountains, the Minshan Mountains, the northern part of the Hengduan Mountains, the Nanling Mountains, the Wuyi Mountains, and the Taiwan Island have the greatest number of species (Figure 2).

The OLS models were used to analyze the relationship between species richness patterns and environmental factors in the Panorpidae. The results revealed that species richness patterns were mainly influenced by Bio18 (Coef = 0.238), elevation (Coef = 0.183), Bio16 (Coef = 0.160), Bio12 (Coef = 0.155), and Bio13 (Coef = 0.148), while negative correlations were discovered with Bio2 (Coef = −0.086) and Bio7 (Coef = −0.072). Although there were some differences, the outcomes of the SARerr model and the OLS models were largely consistent. The species richness pattern was correlated with Bio18 (Coef = 0.087), elevation (Coef = 0.077), Bio14 (Coef = 0.066), Bio12 (Coef = 0.064), and Bio17 (Coef = 0.064), while it was negatively correlated with Bio2 (Coef = −0.064) and Bio15 (Coef = −0.064). Other factors have little relationship with species richness (Table 2).

### 3.2. Evaluation of the MaxEnt Model and the Impact of Environmental Variables

**Figure 3:**
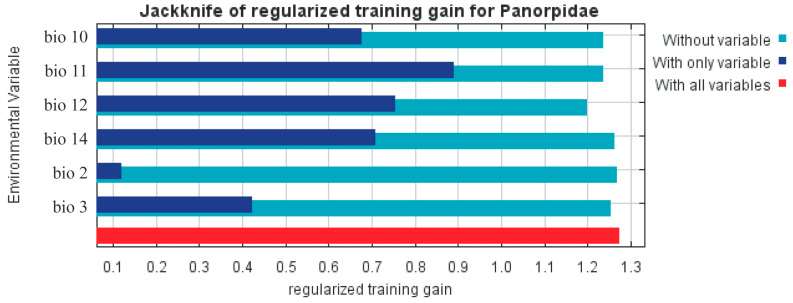
Panorpidae distribution using the jackknife test: significance of key environmental factors.

The results show that the models used in the experiments performed well (AUC = 0.900 and TSS = 0.85). Six major bioclimatic variables were used to create the model: mean diurnal range (Bio2), isothermality (Bio3), mean temperature of warmest quarter (Bio10), mean temperature of coldest quarter (Bio11), annual precipitation (Bio12), and precipitation of driest month (Bio14) (Table 3). Figure 3 shows the Jackknife test results for each environmental variable in the MaxEnt model. Four environmental factors (Bio10, Bio11, Bio12, and Bio14) have relatively high returns (>0.4) when used separately, indicating that they have more important information. However, when utilized alone, Bio2 and Bio3 both offer relatively low contributions, demonstrating that these two environmental factors are less informative. To evaluate the adaptability of Panorpidae’s global distribution, Bio10, Bio11, Bio12, and Bio14 were chosen as significant environmental criteria for this study.

**Figure 4:**
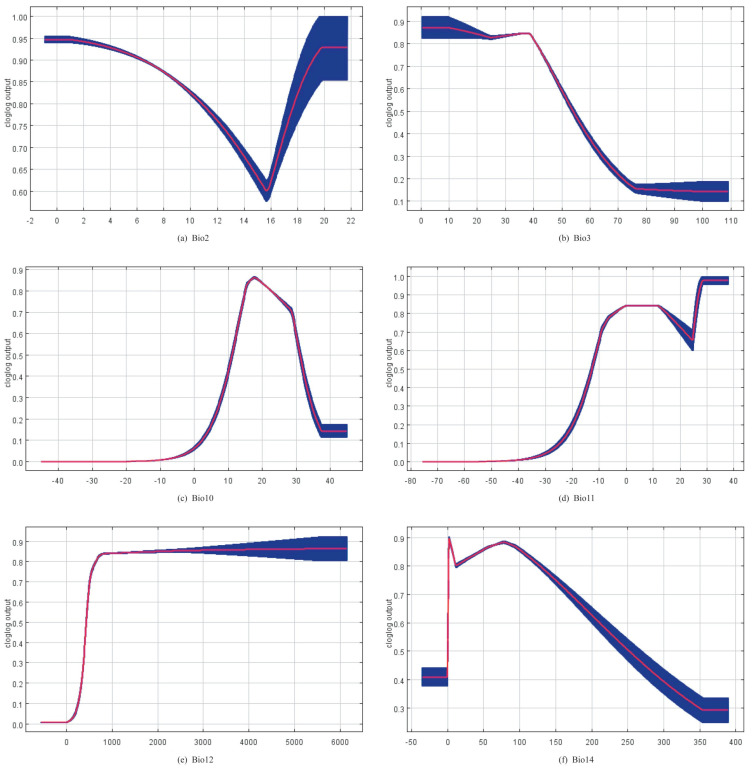
Response curves for the six key bioclimatic factors: (**a**) mean diurnal range; (**b**) isothermality (Bio2/Bio7) (×100); (**c**) mean temperature of warmest quarter; (**d**) mean temperature of coldest quarter; (**e**) annual precipitation; (**f**) precipitation of driest month.

Figure 4 shows the response curves for the six bioclimatic variables obtained from the MaxEnt model. 

### 3.3. Global Distribution of Panorpidae

**Figure 5:**
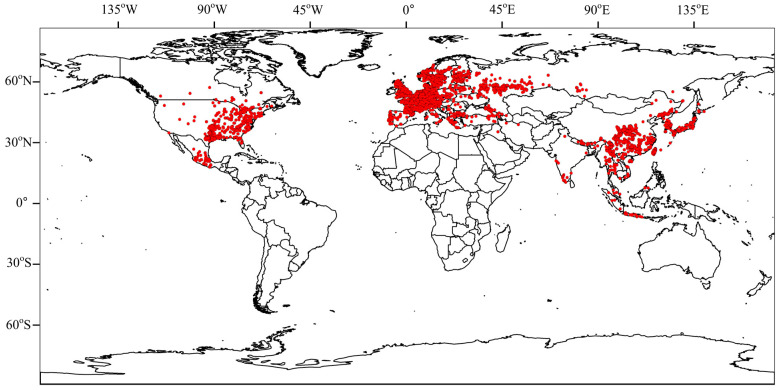
Global distribution of Panorpidae.

The global distribution of Panorpidae is shown in Figure 5. Panorpidae have a distribution range in Asia approximately between 10–50° N and 90–140° E, which includes central, eastern, and southern China and Japan. There is also a small distribution in India, Myanmar, Thailand, Viet Nam, Malaysia, and Indonesia. Panorpidae are primarily found in Europe between the latitudes of 40 and 70° N, which includes western Russia, Germany, Italy, and France. They are primarily found in North America between the latitudes of 30 and 50° N, which includes the eastern United States and southeast Canada.

### 3.4. Predicted Habitat Suitability for Three Historical Eras

**Figure 6:**
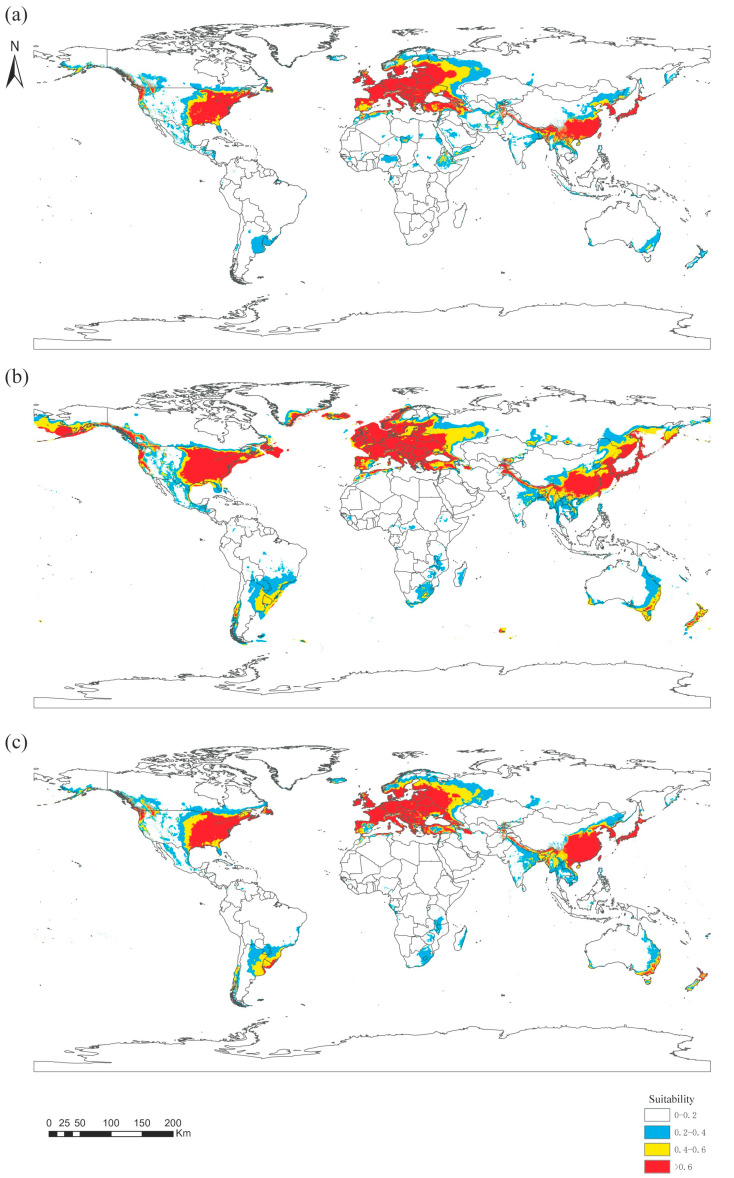
Distribution of the suitable habitat of Panorpidae over three historical periods: (**a**) the Last Interglacial, LIG; (**b**) the Last Glacial Maximum, LGM; and (**c**) 1970–2000, Current.

Based on the maximum entropy model simulation, six major environmental variables and distribution data were used to calculate the global suitable habitat map of Panorpidae in three historical periods (the Last Interglacial, LIG; the Last Glacial Maximum, LGM; and 1970–2000, Current; Figure 6). The suitability of habitat is divided into four levels: highly suitable area, medium suitable area, low suitable area, and unsuitable area. The prediction results show that the highly suitable regions in these three periods are mainly concentrated in Southeast Asia, Western Europe, and eastern North America.

During these three periods, the area of the suitable habitat increased and then decreased as the global temperature rose and then dropped. From LIG to LGM, the area of the suitable habitat for Panorpidae increased significantly, expanding northward into southern Denmark and some coastal areas of the Russian Far East. Meanwhile, the area of suitable habitat in central South America, southeastern Africa, and eastern Australia also increased, although there are no official records of Panorpidae in these areas. Overall, the area of the global suitable habitat (0.2–1) has expanded from 34.14 to 54.09 million km^2^, an increase of 58.4%.

From LGM to Current, as the temperature rose, the area of habitat shrunk (Figure 6). Shrinkage occurred mainly in the eastern part of Europe and northeastern Asia. The area of the global suitable habitat decreased from 54.09 to 37.69 million km^2^, only 69.68% of the LGM period. It is noteworthy that high suitability areas (>0.6) in southeastern Africa and southeastern Australia have instead increased. Comparing the two periods of Current and LIG, the range of the suitable habitat in China has expanded northward, and the area of highly suitable habitat in south-central Asia has decreased. Some areas of low suitability in central Africa and the Indonesian archipelago have disappeared, but some areas in eastern South America, southeastern Australia, and New Zealand have become more suitable (Table 4).

### 3.5. Distribution Trends of Panorpidae during Future Global Warming

The MaxEnt model was used to simulate the suitable habitat of Panorpidae in future global warming scenarios (RCP 4.5) by the 2070s. The regions with a higher possibility of Panorpidae survival are receding due to the changing environment, from 37.69 to 36.55 million km^2^. Highly suitable zones have shrunk slightly in North America, Europe, and Asia. Consequently, overall Panorpidae habitat availability declined with climate warming (Table 4).

**Figure 7:**
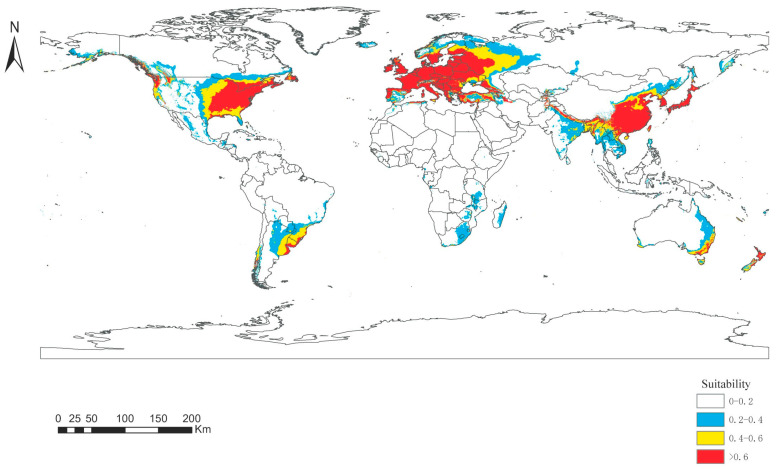
Distribution of the habitat appropriateness of Panorpidae under global warming.

The area of the suitable habitat under the global warming scenarios is more similar to that of the LIG period. However, the distribution of some suitable areas in these two periods differs greatly, mainly from central to southwestern China, Myanmar and eastern India in Southeast Asia, and central and western North America (Figure 7).

## 4. Discussion

### 4.1. Patterns of Species Richness and Influencing Factors

Due to insufficient sampling and restricted distributions of some species, field surveys alone may considerably underestimate species richness [55]. Ecological niche modeling (ENM) can help estimate species distributions under inadequate sampling [56]. It is difficult to comprehensively understand the species richness patterns of Panorpidae because previous studies are scarce and mostly focus on within a specific region [11,12]. In this study, the distribution pattern of Panorpidae on a global scale was analyzed, revealing that most diverse geographic areas are primarily in eastern Asia, Western Europe, and eastern North America. In previous research, China was thought to be the center of the origin and divergence of Panorpidae on a worldwide scale [4,57]. The current discovery of China’s highest species richness supports this hypothesis. The centers of species diversity of Panorpidae in China are mainly located in the Qinling, Bashan, Minshan, Nanling, the northern Hengduan Mountains and Taiwan Island.

Panorpidae migrated from Asia to North America via the Bering land bridge in the Eocene to Oligocene periods [58,59]. During the Middle to Late Miocene, the central and western regions of North America dried out [60], leading to the extinction of many scorpionfly taxa in this area. However, similar groups in the humid eastern regions were able to persist, thus forming the current distribution pattern. The ancestor of Panorpidae was Orthophlebiidae, which appeared relatively late in the entire Mecoptera evolutionary history [61]. After originating in East Asia, Panorpidae expanded their distribution southward [59], but only as far as southern Indonesia, failing to reach the Southern Hemisphere. This also may explain why the species of this family are distributed only in the Northern Hemisphere.

Species richness is spatially characterized [62], and if the spatial autocorrelation phenomenon of these indicators is not taken into consideration, it will affect the accuracy of the assessment about the relationship between species richness and environmental factors. The spatial autocorrelation phenomenon may lead to bias in the regression parameters. Commonly used spatial regression models such as the SAR or OLS models can eliminate more residual spatial autocorrelation and hence have greater explanatory power [62]. In order to study the relationship between species richness and environmental factors, we used OLS and SAR models. The findings indicated that both models essentially reached the same conclusion that species richness had a highly significant positive correlation with factors related to precipitation as well as elevation, suggesting that precipitation and elevation were dominant factors of species richness patterns.

Some Panorpidae were found to have seasonal polyphenism as a way of adapting to their habitat environment, with temperature being the main determinant [63,64]. However, these studies only analyzed the effect of temperature and neglected the precipitation factor. Apart from that, previous studies generally considered that the distribution of scorpionflies is mainly influenced by temperature [10,12]. In contrast to previous studies, our present research discovered that among a number of climatic factors, the richness distribution pattern is primarily influenced by precipitation factors, with temperature factors having a slight effect.

There are few previous studies on the effect of precipitation or humidity on scorpionflies. It has been demonstrated that *Neopanorpa* and *Leptopanorpa* from Java Island had high humidity requirements [65]. As a result, the distribution pattern of Panorpidae in this region is strongly correlated with the local precipitation. Additionally, earlier research indicated that reproduction is influenced by humidity, especially the ovipositional process [66]. We speculate that humidity may also have an impact on how the Panorpidae adapt to their surroundings. Panorpidae can adapt to local temperatures by adjusting their eclosion time, but precipitation can affect the ecology and thus determine whether they can survive in an area. Therefore, Panorpidae’s species richness is mainly related to precipitation.

### 4.2. Relationship between Environmental Factors and the Suitability of Panorpidae Habitat

The mean temperature of the warmest quarter (Bio10), the mean temperature of the coldest quarter (Bio11), the annual precipitation (Bio12), and the precipitation of the driest month (Bio14) were the variables that had the greatest influence on the distribution of Panorpidae among the bioclimatic variables analyzed in this study. The findings indicated that the Panorpidae tended to live in environments where the warmest quarter’s mean temperature ranged from 14 to 26 °C, the coldest quarter’s mean temperature ranged from −8 to 15 °C, the annual precipitation greater than 600 mm, and the driest month’s precipitation less than 80 mm, approximately. The statistical results of these models showed that the Panorpidae preferred the cold and humid habitat, which was consistent with the previous research [4,10,57]. The Panorpidae primarily occurred in the canopy environment with good vegetation coverage, such as forests, shrubs, and canyons in high mountains [67], which may be intimately tied to their origins and evolution [68,69].

Precipitation affects the distribution of insects in both direct and indirect ways. First, it directly affects the living environment of insects. Increased precipitation can create damp environments and shady habitats for insects that are accustomed to the cold, increasing their chances of surviving [70]. Panorpidae are cold-adapted insects, thus an increase in precipitation is likely to make their habitats more favorable. Female adults burrow their abdomens into loose, moist soil to lay eggs [71], making them particularly sensitive to soil temperature and humidity [72]. Soil moisture will fluctuate as a result of precipitation [73]. Additionally, it was discovered that as the average annual precipitation increased, so did the species richness of the Plecoptera [70]. However, excessive precipitation may harm the microenvironment of insects and decrease the survival rate of insect eggs, thereby having a negative impact on their distribution [74]. Therefore, it is hypothesized that excessively dry or moist soil may have an impact on the reproductive process of Panorpidae. Second, by affecting the abundance and type of plants, precipitation indirectly influences how insects are distributed. Increased precipitation encourages plant growth, and the vegetation in return provides habitats and a food source for phytophagous insects [75,76]. The adults and larvae of Panorpidae mainly feed on carrion, but the adults occasionally eat flower berries [77,78,79]. The adult Panorpidae frequently rest on the adaxial surface of leaves. This is likely another factor that may influence how precipitation impacts Panorpidae distribution.

The temperature factors are the second most important influence on the distribution of Panorpidae, next to precipitation factors. Although many habitats are suited for their survival, adult Panorpidae have poor flying abilities so that they are unable to migrate over great distances. The Panorpidae are prone to extreme climatic events, therefore at the end of the glacial cycle, they migrated to higher latitudes and elevations. They typically create sky-island-like distribution patterns at mountaintops, resulting in geographic isolation in the interglacial periods [3]. The processes of migration, isolation, and dispersion of these montane species are useful for studying the biodiversity of the Ice Age refugia, since geographic isolation encourages the speciation process and increases biodiversity. It has been suggested that biodiversity may suddenly and dramatically decline as a result of rising temperatures due to climate change [80,81,82]. In addition, habitat fragmentation caused by climate change is expected to lead to the extinction of montane species and species with restricted distribution [83,84]. According to hypotheses of Li et al., some neotenic Lycidae in Southeast Asia have a discontinuous distribution pattern due to unsuitable habitats caused by climate change [85].

Numerous species, notably insects, will be impacted by the continuous climate warming, since the changes in temperature may have a significant impact on their fundamental physiological processes [86]. Some species may adjust to climatic warming in a variety of ways, such as advancing their phenologies or changing their geographic distributions to follow suitable temperature ranges in order to cope with the changes in the external environment brought on by global warming [87,88,89]. As cold-adapted insect groups, the Panorpidae likely respond to global warming by retreating into refugia in higher mountains where the temperature is moderately cool in the summer season. Consequently, the biodiversity of Panorpidae may be vulnerable to global warming.

### 4.3. Simulation of Habitat Suitability for Three Periods of History

From the LIG to LGM period, the area of suitable habitat increased significantly as temperature decreased. During ice ages, global temperatures dropped, allowing some cold-adapted montane species to shift to the foothills and expand their ranges [16,17,18]. After the end of the ice age, global temperatures gradually increased. These species moved from low-latitude, low-altitude regions to high-latitude, high-altitude regions, and their ranges shrank as a result. This could be one of the causes of the distribution area’s contraction from the Last Glacial Maximum to the modern era [4,10,57]. Previous ecological studies on Panorpidae have tended to focus on only a few species within a genus, such as *Cerapanorpa* and *Dicerapanorpa* [4,10,57]. These studies showed that the potential distribution range of the LGM period had expanded significantly compared to the present day, and is widely and continuously distributed in the Qinling, Minshan, and Bashan Mountains. This coincides with our results that the global potential distribution area of Panorpidae was 43.52% larger during the LGM period than it is today. Due to the dip in temperature during the LGM, the ice sheets grew and sea levels fell, exposing the continental shelf and resulting in a sizable coastal plain [90] (Figure S1). This might have facilitated the expansion of the Panorpidae’s suitable survival area. For instance, the Southeast Asian islands and the Asian plate were united during the ice age, but after it ended, they separated into archipelagic regions [91], reducing the continental area. It is clear that changes in sea level and exposure of the continental shelf also have a significant impact on how species are distributed.

The suitable survival area of Panorpidae dropped from LGM to 1970–2000. Dramatic population growth, environmental devastation, the acceleration of urbanization, and other factors have altered the earth’s climate significantly, resulting in a number of important ecological problems [92,93,94,95]. In our study, the distribution range of Panorpidae’s highly suitable survival area has decreased as a result of climate change. According to Meulebrouck et al., numerous wildland species in Western Europe have suffered major decline in recent decades as a result of habitat loss [96]. Thomas (2004) demonstrated that over the past few decades, annual global climate change has changed the distribution and abundance patterns of many species, and that we are likely to experience a species mass extinction event [80]. Climate change may have resulted in the extinction of the poorly dispersing lineage in net-winged beetles, especially when they are restricted to small forest refugia [68].

### 4.4. Changes in Panorpidae Distribution in the Future

The increase in global temperature is expected to continue and could reach 1.4–5.8 °C by 2100 [97]. According to predictions made by global warming models, roughly 24% of terrestrial species will likely be “declared extinct” in the near future [80]. Other studies indicated that 20–30% of plants and animals will go extinct if the average global temperature rose by 2–3 °C; if it rose by more than 4 °C, a substantial number of organisms would perish, destroying the entire planetary ecosystem [97]. Certainty, cold-adapted species, insects in particular, are more vulnerable to global warming. In fact, insects are ectotherms, so that their tiny population sizes and constrained habitat ranges are heavily influenced by environmental temperatures, as indicated in studies of fossils [98,99]. The findings based on the MaxEnt model indicate that the global warming scenarios would result in a lesser amount of suitable habitat for Panorpidae than currently available. Obviously, global warming may be detrimental to the survival of Panorpidae.

On the other hand, global warming has the potential to degrade the habitat environment of the species, likely influencing insect development, growth, and mortality [100]. The survival and reproduction of insects are affected by habitat changes [101,102]. Climate change can alter the survival strategies of insects and also have an impact on their distribution. Our findings indicate that Panorpidae migrate to higher latitudes and elevations as a result of rising temperatures, which may significantly reduce their habitat areas. In addition, there is the migration of some insects and plants to higher altitudes and higher latitudes in response to global warming. For instance, due to the grasshopper’s high cold tolerance, it is expected that it would migrate northward during global warming [103]. Similar studies in Europe have shown that the migration of butterfly species is northward [104]. Additionally, following an increase in temperature, the distribution of thermophilic plants has been shown to shift toward higher latitudes and elevations [105,106]. However, the productivity of the high-altitude area is lower than that of the middle-altitude zone, and the suitable habitat area is becoming smaller [107,108,109,110,111]. Many individuals may be lost in the process of moving to higher altitudes, and the population size will gradually decrease, eventually resulting in the loss of species richness and genetic diversity. Therefore, to reduce the adverse effects of climate change on Panorpidae, we propose to establish nature preserve areas in their biodiversity centers.

In response to climate change, species often use both migration and adaptation strategies to adapt to their environment [112]. Additionally, species adjust to climate change on three different scales: temporal, spatial, and self-change scales [113]. Panorpidae most likely adjust to their environment through changes in temporal and spatial scales. Different Panorpidae species from different regions have varying eclosion times and can survive at different latitudes, which may be an adaptive response to the environment. Numerous studies imply that allochronic divergence is connected to species’ environmental adaptability, resource utilization, or temperature adaptation [114,115,116,117,118]. As previously reported in butterflies, allochronic divergence in insects is directly associated to temperature acclimation [119]. On the other hand, because of their short life cycles, insects have the potential to adapt to different environments. In a study on the evolutionary adaptation of scorpionflies, *Panorpodes paradoxus* evolved an “alpine type” to adapt to the low-temperature environment [120]. This is a result of Panorpidae’s adaptation to the uplift of Japanese mountains and continuous ice–interglacial cycle.

The present study also found that although the area of suitable habitats for Panorpidae was correspondingly reduced in the future, the main distribution areas of suitable habitats did not move much, as in the case of previous studies [121,122,123].

### 4.5. Implications for Biological Conservation

Understanding the probable ranges of rare species under present or forecast climatic scenarios is essential for species conservation [124,125]. There are two processes leading to species extinction: reduction in geographic distribution (large scale) and decline in population abundance (local scale) [126]. However, these rare species often lack distribution data, which makes it difficult to designate appropriate conservation methods [127]. In this regard, new developments in ecological modeling have improved our ability to estimate and predict species distributions and can compensate for the lack of available data in species conservation [128]. The MaxEnt model was used in this work to assess the possible distribution of Panorpidae under several climate scenarios. The findings demonstrated that suitable areas of Panorpidae did not significantly change under global warming temperature scenarios, consistent with the relatively limited migration capacity of Panorpidae. Nevertheless, the correct microhabitats can allow some lineages to persist for a very long time. In other words, we advocate more attention to highly suitable areas and conservation measures in these areas [129]. Our predicted results likely provide information for the present population trends, and may have an impact on further studies and conservation initiatives [128].

## 5. Conclusions

In this study, we investigated the distribution pattern of Panorpidae and the relationship between their species richness and environmental factors. High species richness areas are mainly located in south-central China. The main factors affecting species richness are those related to precipitation as well as elevation. Other environmental factors, however, had comparatively little impact.

Using the best MaxEnt model measured by the AUC and TSS index, the probable survival area of Panorpidae was examined and predicted. The areas suitable for Panorpidae are mainly distributed in southeastern North America, Europe, and southeastern Asia. Six major bioclimatic factors that influence the distribution of species—mean diurnal range (Bio2), isothermality (Bio3), mean temperature of warmest quarter (Bio10), mean temperature of coldest quarter (Bio11), annual precipitation (Bio12), and precipitation of driest month (Bio14)—are chosen from a total of 19 bioclimatic factors. From LIG to LGM to Current, the area of the suitable habitat increased and then decreased. For cool-adapted insects such as scorpionflies, the range of the suitable habitat is maximum in the LGM period. According to the simulation result in global warming scenarios, the habitat areas of Panorpidae decreased as a result of increased temperature. This indicates a decline in population size and genetic diversity in the future for Panorpidae.

In addition to the aforementioned bioclimatic factors and elevation, there are many factors that may also have an impact on the suitable habitat of scorpionflies, such as vegetation cover, soil conditions, and human activities. In subsequent studies, these factors can be considered for analysis and are able to predict the distribution of Panorpidae more precisely. Furthermore, a defect of ecological niche models is that they rely strongly on the quality of data records, which are often influenced by artificial randomness.

## Figures and Tables

**Table 1:** Bioclimatic variables utilized in the model and the proportional contributions of 19 bioclimatic factors to the Maxent model for Panorpidae.

Variables	Description	Percent Contribution
Bio1	Annual mean temperature	6.1
Bio2	Mean diurnal range (mean of monthly (max temp-min temp))	1.4
Bio3	Isothermality (Bio2/Bio7)(×100)	2.3
Bio4	Temperature seasonality (standard deviation ×100)	3.5
Bio5	Maximum temperature of warmest month	0.6
Bio6	Minimum temperature of coldest month	8.1
Bio7	Temperature annual range (Bio5-Bio6)	0.2
Bio8	Mean temperature of wettest quarter	0.4
Bio9	Mean temperature of driest quarter	0.2
Bio10	Mean temperature of warmest quarter	1.3
Bio11	Mean temperature of coldest quarter	15.1
Bio12	Annual precipitation	46.3
Bio13	Precipitation of wettest month	0.1
Bio14	Precipitation of driest month	13.3
Bio15	Precipitation seasonality (coefficient of variation)	0.2
Bio16	Precipitation of wettest quarter	0.1
Bio17	Precipitation of driest quarter	0.2
Bio18	Precipitation of warmest quarter	0.2
Bio19	Precipitation of coldest quarter	0.3

**Table 2:** Correlations between species richness and environmental factors calculated by spatial error simultaneous autoregressive (SARerr) model and ordinary least squares (OLS) model. Coef, coefficient of regression; R^2^ (%), determination coefficient for the OLS model; and pseudo R^2^ (%), determination coefficient for the SARerr model.

	Species Richness			
	Coef_OLS_	R^2^_OLS_	Coef_SAR_	Pseudo R^2^_SAR_
Bio1	0.043	0.098 ns	0.003	52.538 ns
Bio2	−0.086	0.660 **	−0.064	52.385 **
Bio3	−0.010	0.009 ns	0.018	53.273 ns
Bio4	−0.056	0.232 ns	−0.040	52.893 ns
Bio5	−0.027	0.007 ns	−0.057	52.977 *
Bio6	0.054	0.201 ns	0.028	53.071 ns
Bio7	−0.072	0.427 *	−0.056	53.088 *
Bio8	0.049	0.158 ns	−0.041	52.676 ns
Bio9	−0.031	0.096 ns	−0.006	52.744 ns
Bio10	0.020	0.040 ns	−0.043	52.637 ns
Bio11	0.048	0.139 ns	0.021	53.074 ns
Bio12	0.155	2.318 ***	0.064	51.806 **
Bio13	0.148	2.091 ***	0.030	51.205 ns
Bio14	0.013	0.017 ns	0.066	51.552 **
Bio15	0.036	0.042 ns	−0.064	51.482 **
Bio16	0.160	2.480 ***	0.040	51.141 ns
Bio17	0.008	0.006 ns	0.064	51.625 **
Bio18	0.238	5.581 ***	0.087	50.743 ***
Bio19	−0.072	0.4291 *	0.023	52.439 ns
Elevation	0.183	3.246 ***	0.077	51.563 **

*** *p* < 0.001; ** *p* < 0.01; * *p* < 0.05; ns, not significant (*p* > 0.05).

**Table 3:** Environmental factors used for MaxEnt modeling the habitat suitability distribution of Panorpidae.

Data Source	Category	Variables	Abbreviation	Units
Panorpidae Worldclim	Bioclimatic	Mean diurnal range	Bio2	°C
		Isothermality (Bio2/Bio7)(×100)	Bio3	%
		Mean temperature of warmest quarter	Bio10	°C
		Mean temperature of coldest quarter	Bio11	°C
		Annual precipitation	Bio12	mm
		Precipitation of driest month	Bio14	mm

**Table 4:** The area of Panorpidae’s four habitat suitability distribution over four eras.

Habitat Suitability	Historical Periods			Global Warming Scenarios
	Last Interglacial	Last Glacial Maximum	1970–2000	2070s RCP4.5
	Area (million km^2^)	Area (million km^2^)	Area (million km^2^)	Area (million km^2^)
0–0.2	114.7584	94.8089	111.2099	112.3447
0.2–0.4	13.1229	16.8937	13.2336	12.8701
0.4–0.6	6.5181	14.5506	8.3257	7.9061
>0.6	14.5005	22.6467	16.1308	15.7791

## Data Availability

The data presented in this study are available on request from the corresponding author.

## Supplementary Materials

The following supporting information can be downloaded at: https://www.mdpi.com/article/10.3390/insects14040332/s1, Figure S1: Coastline expanded during LGM.
