# Supplementary figures and images for: The Distribution Pattern and Species Richness of Scorpionflies (Mecoptera: Panorpidae)
Source: Insects. 2023 Mar 29;14(4):332. doi: 10.3390/insects14040332 (PMC10146745; doi:10.3390/insects14040332)

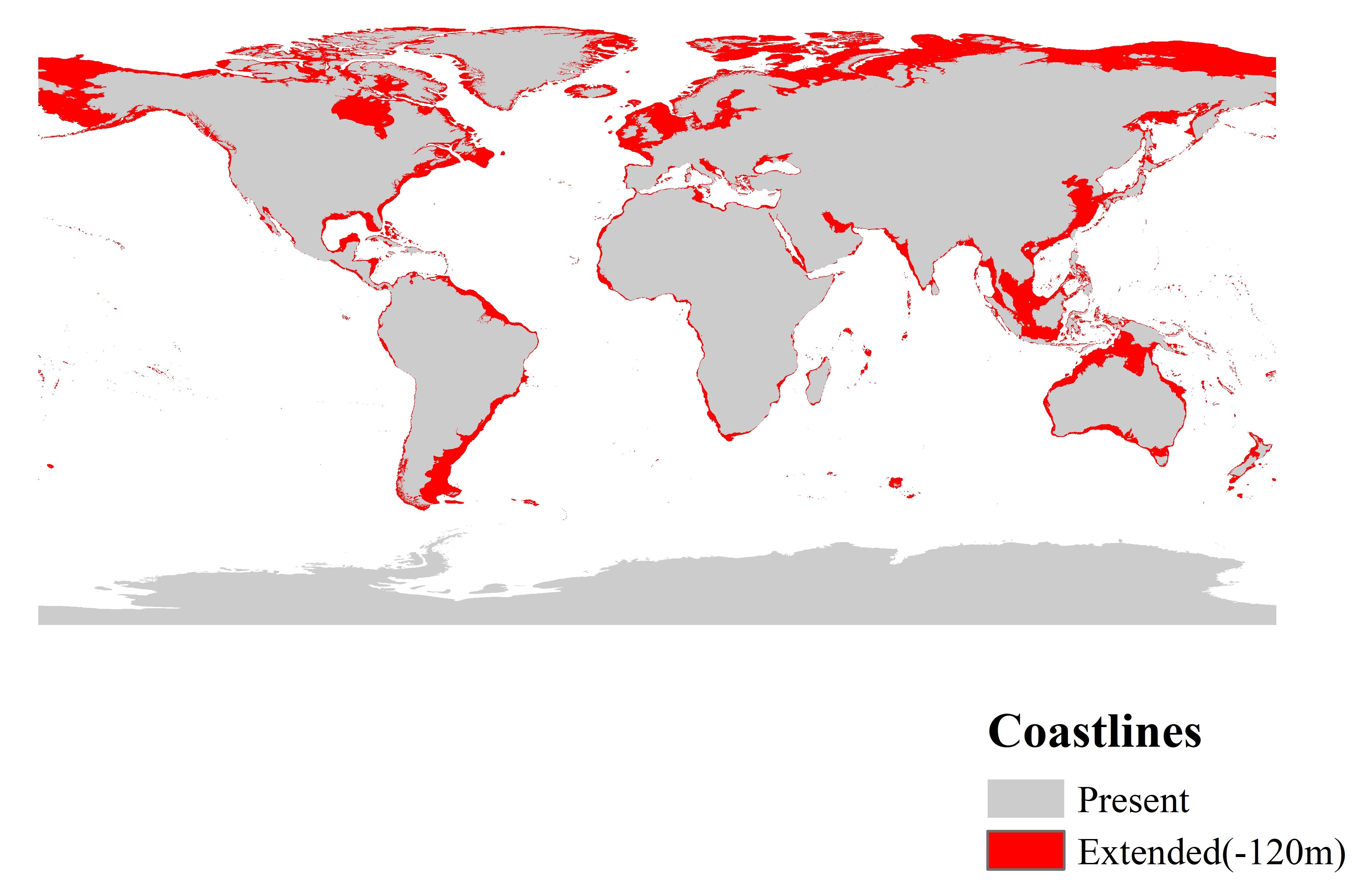

Supplement: Supplementary file 1 [file insects-14-00332-s001.zip › Figure S1.jpg]
